# Pacemaker Malfunction Triggered by Cybersecurity Activation During Carbon Ion Radiotherapy: A Case Report

**DOI:** 10.7759/cureus.83835

**Published:** 2025-05-10

**Authors:** Eisuke Horigome, Nobuteru Kubo, Makoto Sakai, Hiroshi Hasegawa, Tatsuya Ohno

**Affiliations:** 1 Department of Radiation Oncology, Gunma University Graduate School of Medicine, Maebashi, JPN; 2 Department of Cardiovascular Medicine, Gunma University Graduate School of Medicine, Maebashi, JPN

**Keywords:** carbon ion radiotherapy, cardiac implantable electronic device, cybersecurity function, high-energy neutron, malfunction, pacemaker

## Abstract

Radiation therapy for patients with cardiac implantable electronic devices (CIEDs) poses a unique risk of device malfunction. Carbon ion radiotherapy (CIRT) is particularly effective against tumors that are resistant to conventional therapies or located near critical organs owing to its high linear energy transfer and superior biological effectiveness. Conversely, neutrons generated during CIRT may interfere with CIEDs, potentially causing malfunction. Although numerous studies have reported device malfunctions related to X-ray and proton beam therapies, only a few case reports have described similar issues during CIRT, indicating that such risks, while rare, should not be overlooked.

Herein, we report a case with early-stage lung cancer and an implanted pacemaker who experienced device malfunction during CIRT.

An 83-year-old male patient, clinically diagnosed with cT1bN0M0 Stage IA2 lung cancer in the left upper lobe, was treated with CIRT. Despite careful treatment planning to minimize neutron exposure by selecting beam directions that maximized the distance between the device and the beam path, the pacemaker’s cybersecurity function, which is designed to prevent unauthorized access by locking external communication and placing the device into a failsafe backup mode, was unexpectedly activated, disrupting communication with its external programmer. The malfunction was hypothesized to result from the high-energy neutrons generated during radiation delivery. The device malfunction was detected immediately before the final (fourth) fraction was delivered. A three-day interval, including a weekend and holiday, occurred between the third and fourth fractions due to scheduling. Although electrocardiography findings remained stable during this period, a causal relationship between the delay and the malfunction could not be excluded. Consequently, CIRT was discontinued after three fractions with a total dose of 45 Gy (relative biological effectiveness or RBE). This decision was based not only on the device malfunction but also on concerns about the ability to recover device function if a second malfunction occurred. In addition, 45 Gy (RBE) was considered sufficient for tumor control in this clinical setting. The patient remained asymptomatic and in stable general condition after CIRT and was discharged. As the patient resided in a remote area, follow-up was entrusted to the referring physician. No subsequent reports of device abnormalities or reprogramming needs have been received.

This case highlights the potential for unforeseen software errors in CIEDs during CIRT, emphasizing the need for continuous risk evaluation and multidisciplinary management. At our institution, we have standardized a comprehensive protocol to ensure device safety during radiation therapy, including pretreatment evaluation by cardiologists, continuous electrocardiogram monitoring during the treatment period, and device interrogation before and after each session of irradiation. This management system enabled the prompt detection and resolution of this issue. Future research should focus on optimizing radiation treatment planning, improving device software robustness, and exploring shielding strategies to enhance safety in CIED-equipped patients receiving particle therapy.

## Introduction

Radiation therapy, a fundamental treatment for cancer, contributes to effective local control either as a standalone approach or in combination with surgery, chemotherapy, or immunotherapy [1,2]. Although conventional photon-based radiation therapy is widely used, particle therapies such as proton beam therapy (PBT) and carbon ion radiotherapy (CIRT) have become increasingly prevalent owing to their improved dose distribution and reduced healthy tissue exposure [3]. CIRT is characterized by high linear energy transfer (LET) and superior biological effectiveness. It is also effective in treating tumors that are resistant to conventional radiation therapy or located near critical healthy organs and tissues [4]. Recent large-scale registry studies have also demonstrated favorable outcomes of CIRT in patients with early-stage lung cancer [5].

Radiation therapy, including particle therapy, poses unique risks to patients with cardiac implantable electronic devices (CIEDs) such as pacemakers or implantable cardioverter-defibrillators; these include device resets, inappropriate mode switching, and complete device failure [6]. Neutron production associated with PBT and CIRT can interfere with CIEDs, potentially leading to malfunctions that compromise patient safety. These malfunctions are primarily caused by single-event effects such as bit flips in memory circuits, induced transient currents, and software errors resulting from interactions with high-energy neutrons. Although some mitigation strategies have been proposed - such as increasing the distance between the device and the beam axis, using beam angle optimization, and selecting active scanning techniques when available - these measures have limitations, and standard guidelines specific to CIRT are currently lacking.

Although the risks of CIED malfunction during photon therapy and PBT are well-documented [7,8], the effects of CIRT on such devices have not been extensively studied. While a few reports of CIED malfunctions during CIRT do exist, they remain rare and have not previously included cybersecurity-related incidents. Given its high LET and distinct physical properties, CIRT presents unique challenges in the management of patients with CIEDs. This case report describes a rare instance of pacemaker malfunction during CIRT for lung cancer, wherein the cybersecurity function of the device was unexpectedly activated, disrupting communication with an external programmer. To the best of our knowledge, this is the first reported case of such an occurrence during CIRT.

## Case presentation

**Figure 1:**
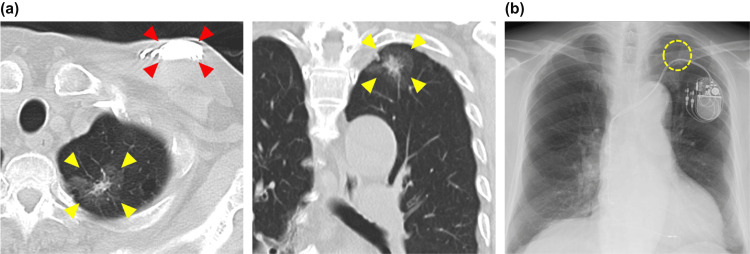
Pretreatment imaging studies (a) Computed tomography (CT) scan. The yellow arrow indicates the left upper lobe lung cancer. The lesion comprises a central solid component with a maximum diameter of 1.2 cm, surrounded by a ground-glass opacity. The red arrow indicates the pacemaker. (b) Chest X-ray. The yellow dashed line shows the left upper lobe lung cancer lesion (not clearly identifiable on radiography but determined based on CT imaging). The tumors and pacemakers were relatively close to each other.

An 83-year-old male patient was clinically diagnosed with left upper lobe lung cancer (cT1bN0M0 Stage IA2 according to the Union for International Cancer Control Eighth Edition) (Figure 1a). The patient had a history of complete atrioventricular block and a magnetic resonance imaging-conditional pacemaker (ZENEX™ MRI, model PM2282; Abbott Cardiovascular, Plymouth, MN, USA) implanted in the left chest (Figure 1b). The device was set in the dual-chamber pacing (DDD) mode at a basic rate of 60-120 bpm. Following a discussion with the institutional cancer board, surgery was deemed feasible. However, the patient refused surgical intervention, and CIRT was selected as the treatment modality. Pretreatment device interrogation was conducted in the cardiology department; the atrial and ventricular pacing rates were 23% and 99%, respectively, indicating high pacemaker dependency. Consequently, inpatient CIRT was recommended. Continuous electrocardiography (ECG) monitoring and device interrogation were planned before and after each radiation fraction. After treatment, cardiological evaluation would determine discharge feasibility.

**Figure 2:**
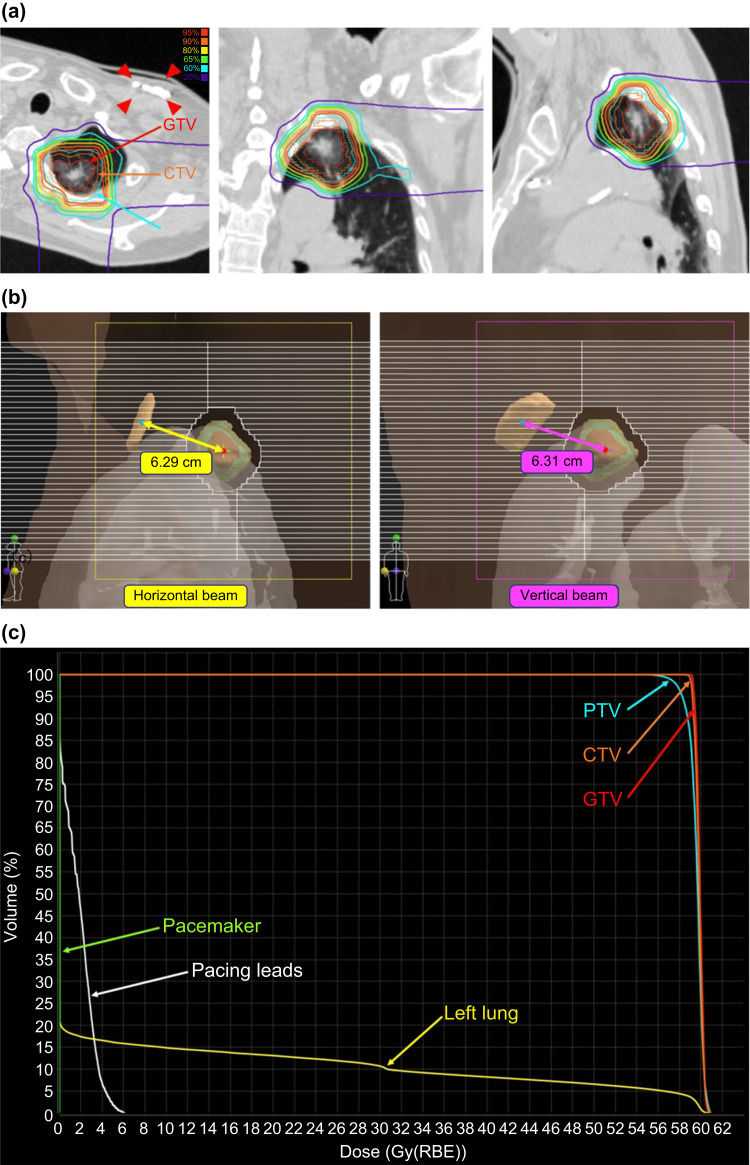
Carbon ion radiotherapy: total dose of 60 Gy (relative biological effectiveness (RBE)) in four fractions (a) Dose distribution. Red: GTV; orange: CTV; cyan: PTV. The red arrow indicates the pacemaker. The treatment plan involved tilting the treatment couch 20° counterclockwise and delivering a horizontal beam in the supine position and a vertical beam in the prone position (the beam angles on the simulation CT were 70° and 160°, respectively). (b) BEV of each beam. The yellow cross indicates the isocenter, and the red dot represents the beam axis passing through the isocenter (appearing as a dot due to the BEV, overlapping with the isocenter). The cyan dot marks the center of the pacemaker, whereas the light-brown structure represents the pacemaker itself. By selecting beam directions that maximized the distance between the beam axis and the center of the pacemaker, the distance was 6.29 cm for the horizontal beam and 6.31 cm for the vertical beam. (c) Dose-volume histogram (DVH). Red: GTV; orange: CTV; cyan: PTV; yellow: left lung; white: pacing lead; green: pacemaker GTV: gross tumor volume; CTV: clinical target volume; PTV: planning target volume; BEV: beam’s eye view

Treatment was planned to minimize pacemaker malfunction while ensuring effective dose delivery to the target. The beam angles were carefully optimized to maximize the distance between the device and the beam axis. Two beam paths were selected: a horizontal beam at 70° in the supine position and a vertical beam at 160° in the prone position, with two fractions delivered for each path. The treatment plan followed institutional protocols with a total dose of 60 Gy (relative biological effectiveness (RBE)) in four fractions. Treatment was planned using the XiO-N system (Elekta, Stockholm, Sweden), ensuring that the device was positioned as far as possible from the beam axis to minimize neutron exposure (Figures 2a-2b). Dose calculations with the XiO-N system estimated the maximum dose to the pacemaker at 0.00 Gy and that to the pacing leads at 6.06 Gy (Figure 2c). Although the possibility of additional physical shielding was considered, no practical shielding options could be implemented due to the tumor and pacemaker being located at approximately the same axial level. Therefore, optimization of beam direction and treatment geometry was prioritized as the primary mitigation strategy.

The initial three fractions (45 Gy (RBE)) were delivered without complications. ECG monitoring during treatment showed no abnormalities; device interrogations before and after the first three fractions revealed no issues. During each device interrogation before and after the first three fractions, the following parameters were evaluated: pacing thresholds, lead impedance, atrial and ventricular sensing values, programmed pacing rates, and battery status. In addition, real-time ECG monitoring was used to assess pacing function and waveform morphology throughout the treatment period, and no abnormalities were identified. A three-day interval, including a weekend and holiday, elapsed between the third and fourth fractions, during which ECG findings remained stable. However, device interrogation before the final fraction revealed a loss of communication with the pacemaker. ECG monitoring confirmed continued pacing, with the heart rate maintained at approximately 60 beats/minute. Consultation with Abbott Medical Japan LLC revealed that the pacemaker’s cybersecurity function had been activated, placing the device in backup mode. Although the precise trigger remains uncertain, the malfunction was presumed to result from secondary neutron exposure affecting the device’s telemetry or communication circuits. As a result, the maximum tracking rate was reset to 130 bpm, and various alert and response settings, including those for atrial fibrillation and high ventricular rates, were disabled. Additionally, both the maximum tracking rate and the maximum sensor rate settings were found to have reverted to their default values, consistent with the device entering backup mode. Abbott representatives deactivated the cybersecurity function and restored the original settings. Cardiology evaluation confirmed that device replacement was unnecessary. However, due to uncertainty regarding the ability to recover the pacemaker in the event of a repeated malfunction during the final fraction, cardiologists recommended discontinuation of CIRT; the radiation oncology department subsequently reevaluated the treatment plan.

Considering clinical experience with stereotactic body radiation therapy (SBRT) for stage I lung cancer, tumor control was considered achievable with 45 Gy (RBE) in three fractions of CIRT. This was supported by comparison to a standard SBRT regimen of 48 Gy in four fractions, with a biologically effective dose (BED) exceeding 100 Gy in both schedules. Furthermore, surgical intervention was still regarded as a viable salvage option in the event of recurrence [9,10]. Thus, treatment was concluded with 45 Gy (RBE) in three fractions. The patient remained asymptomatic and in stable general condition and was thereafter discharged. Due to residence in a remote area, the patient was referred back to the original hospital for follow-up. No reports of tumor recurrence or device abnormalities have been received to date.

## Discussion

CIRT is a potentially effective treatment for patients with cancer and CIEDs. However, it carries a risk of device malfunction [11]. Eliminating this risk is challenging and requires careful management during treatment. In this case, the pacemaker’s cybersecurity function was unexpectedly activated during CIRT, disrupting communication with its external programmer. This incident underscores the possibility of unforeseen effects of radiation therapy on the complex software used by CIEDs, highlighting the need for continuous risk evaluation in patients with such devices and undergoing CIRT.

Neutron production by passive carbon-ion beam irradiation has been documented, with Monte Carlo simulations identifying collimators as the primary sources [12]. High-energy neutrons (>10 MeV), which are predominantly produced in this treatment, interfere with CIED circuits, semiconductors, and internal memory components, increasing the likelihood of malfunction [13]. The frequency of malfunctions decreases with increasing distance from the irradiation center [14]. However, completely protecting devices located near the radiation field is challenging. Treatment plans for CIED-equipped patients undergoing CIRT must be designed considering these risks.

Using the XiO-N system, we estimated the maximum dose for the pacemaker at 0.00 Gy (Figure 2c). Additionally, using the Particle and Heavy Ion Transport code System (PHITS) version 3.32, we estimated the maximum dose for the pacemaker at 0.13 Gy [15]. Although the guidelines for X-ray therapy recommend limiting the dose to CIEDs below 1-2 Gy [16], the calculated dose was far below this range, confirming adequate protection of the pacemaker.

A soft error is a memory bit flip that does not directly correspond to device malfunction. The probability of memory bit flips varies depending on factors, such as manufacturing. Device malfunctions occur only when critical memory components essential for device operation are affected. To evaluate the number of soft errors, we developed a soft error measurement device using a field-programmable gate array [14]. The number of soft errors counted at the pacemaker location was estimated using PHITS version 3.32 [14,15]. Despite the infeasibility of quantitatively estimating the likelihood of pacemaker malfunction, the number of soft errors measured using this device provides a relative risk assessment. The simulations revealed that the total number of soft errors for the treatment plan was 21.8, with 16.4 errors attributed to the horizontal beam and 5.4 errors to the vertical beam. The higher number of soft errors for the horizontal beam is attributable to the higher energy of the carbon-ion beam, which increases the production of high-energy neutrons. High-energy neutrons generated by the horizontal and vertical beams were calculated at 6.0 × 10^7^ and 2.2 × 10^7^ neutrons/fraction, respectively. The PHITS calculations estimated the effective neutron doses at the pacemaker location at 2.2 × 10^-2^ Sv/fraction for the horizontal beam and 6.4 × 10^-3^ Sv/fraction for the vertical beam.

In this case, device interrogation before the fourth radiation fraction detected a communication failure with the pacemaker. The planned sequence for the four fractions was horizontal, vertical, horizontal, and vertical beams, with the first three fractions delivered as scheduled. Considering the higher number of soft errors estimated for the horizontal beam, the abnormalities detected before the fourth fraction aligned with the PHITS simulation results. Device interrogation after the third fraction revealed no abnormalities, with unclear timing between the third and fourth fractions. ECG monitoring during this interval revealed a normal heart rate and no anomalies. Because device abnormalities may not always be detectable immediately after treatment, short-term follow-ups (days to weeks) may be necessary for high-risk cases.

Analysis by cardiologists and the device manufacturer confirmed that the pacemaker’s cybersecurity function was activated, transitioning the device into backup mode. Cybersecurity functions have been developed in response to the increasing adoption of remote monitoring in CIEDs, addressing concerns over cyberattacks that could compromise patient safety by intercepting communication or altering device settings [17]. Although the exact mechanism remains unclear, the activation was most likely caused by high-energy neutron exposure that interfered with internal circuitry and triggered the device’s protective cybersecurity response. In the pacemaker used in this case, the cybersecurity function was triggered by external factors such as the detection of unauthorized communication signals, resulting in a password-locked state and device isolation from all external connections, where only the device manufacturer could disable the lock. This is the first documented case of cybersecurity activation caused by a CIRT. Through close collaboration between the radiation oncology team, cardiologists, and device manufacturers, the root cause was promptly addressed, and treatment was modified and completed without the patient experiencing any symptoms. This outcome underscores the importance of a robust multidisciplinary management system to ensure the safety of patients with CIEDs and those undergoing radiation therapy.

Treatment for this case was concluded at a cumulative dose of 45 Gy (RBE) in three fractions, based on three key considerations. First, cardiologists determined that if a similar malfunction occurred during the fourth fraction, pacemaker functionality might not be restored. Second, due to the peripheral location of the tumor, surgery can remain an option in the event of local recurrence. Finally, tumor control was expected to be achievable using 45 Gy (RBE) in three fractions. For stage I lung cancer, a representative prescription dose for SBRT is 48 Gy in four fractions [9]. Although direct comparison is challenging owing to differences in dose units (Gy for X-rays and Gy multiplied by RBE for carbon ions), the two regimens were evaluated using a linear quadratic model. This model defines the BED as

$\text{BED} = nd \left(1 + \frac{d}{\alpha/\beta} \right)$

where n is the number of fractions, d is the dose per fraction, and α/β is assumed to be 10 for tumor irradiation [18,19]. For stage I lung cancer treated with SBRT, a BED of > 100 Gy is an indicator of favorable outcomes [19]. Using this formula, the BED for X-ray 48 Gy in four fractions was 105.6 and 112.5 for carbon ion 45 Gy (RBE) in three fractions. As the carbon-ion regimen satisfied the BED > 100 Gy criterion and exceeded the BED for the X-ray regimen, sufficient tumor control was anticipated. Thus, the risk of delivering the fourth fraction outweighed its potential benefit, leading to treating the patient with 45 Gy (RBE) in only three fractions.

At our institution, patients with CIEDs who are undergoing radiation therapy are managed according to a protocol that aligns with established guidelines [20]. Following the initial consultation with the radiation oncology department, a device interrogation is conducted by the cardiology team. After patient pacemaker dependency and concurrent arrhythmias are assessed, a management plan for the treatment period is established. During treatment, patients are managed as inpatients with continuous ECG monitoring. Cardiologists or clinical engineers evaluate device functionality before, during, and after each radiation fraction based on the management plan. After the final fraction, cardiologists evaluate the device prior to the patient’s discharge; if no issues are identified, the patient is discharged. This protocol minimizes the risk of device malfunction during the treatment period and facilitates prompt and appropriate responses if malfunctions occur.

## Conclusions

This case highlights the importance of meticulous treatment planning, regular device monitoring, and thorough multidisciplinary collaboration for administering CIRT to patients with CIEDs. Although the risk can be mitigated in part by maximizing the distance between the beam axis and the device, this approach alone may be insufficient, especially for software-level disruptions. In this case, a rare and possibly unprecedented event occurred in which a cybersecurity function was triggered - potentially due to neutron-induced electronic interference. To the best of our knowledge, there are no prior reports of cybersecurity activation in CIEDs during radiation therapy, underscoring the need for further investigation.

Future treatment strategies may benefit from pretreatment evaluations of cybersecurity functions, collaboration with device manufacturers to explore alternative programming or protective modes, and continued exploration of shielding techniques to reduce neutron exposure. Additionally, refinements in soft error modeling and long-term follow-up studies are warranted to better understand both immediate and delayed device responses. Further research in these areas is essential to improve safety and optimize management protocols for CIED-equipped patients undergoing advanced particle therapies.
